# Audit on the documentation of ethnicity within CAMHS

**DOI:** 10.1192/bjo.2021.304

**Published:** 2021-06-18

**Authors:** Charlotte Scott, Roger Lakin

**Affiliations:** Tees, Esk and Wear Valleys Foundation NHS Trust

## Abstract

**Aims:**

Evidence suggests children from minority ethnic groups have lower rates of referrals from primary care to CAMHS, are more likely to be referred via involuntary or compulsory routes and less likely to have access to therapies than children from white backgrounds. In order to understand how ethnicity influences individuals and ensure service innovation meet these needs data collected have to be accurate. The Mental Health Services Data Set outlines all children and families receiving NHS care should have ethnicity included as a mandatory data submission and services are performance managed on this.

The aim of this audit to review the documentation of ethnicity for service users in CAMHS. We agreed that 100% of patients within York and North Yorkshire (Y&NY) CAMHS should have their ethnicity documented.

**Method:**

Integrated Information Centre (IIC) was used to collect data on the documentation of ethnicity for patients under Y&NY CAMHS on 27th August 2020.

**Result:**

The total caseload was 4109 patients.

823 (20%) had their ethnicity documented as ‘unknown’ (the clinician had entered ‘unknown’ or the patient has ‘declined to disclose’).

49 (1.2%) patients had no entry regarding ethnicity (missing).

**Conclusion:**

We recommend further exploration to consider why 1 in 5 patients have ‘unknown ethnicity’ documented. We recommend: conducting a refined search considering the percentage of ‘declined to disclose’ and ‘not stated’ within the ‘unknown ethnicity’ section emailing care coordinators for patients with ‘unknown ethnicity’ or ‘missing ethnicity’ conducting a questionnaire to gather the opinions and experiences of clinicians, patients and families when talking about ethnicity

reviewing the process for documenting ethnicity to improve accuracy developing staff training, to promote a culture of confidence and curiosity when discussing ethnicity Following this intervention we will aim to re-audit and consider if this has improved the rates of documentation of ethnicity.

